# Predicting effects on oxaliplatin clearance: *in vitro*, kinetic and clinical studies of calcium- and magnesium-mediated oxaliplatin degradation

**DOI:** 10.1038/s41598-017-04383-4

**Published:** 2017-06-22

**Authors:** Catherine H. Han, Prashannata Khwaounjoo, Andrew G. Hill, Gordon M. Miskelly, Mark J. McKeage

**Affiliations:** 1 Department of Pharmacology and Clinical Pharmacology and Auckland Cancer Society Research Centre, School of Medical Sciences, Faculty of Medical and Health Sciences, University of Auckland, Auckland, New Zealand; 2 Regional Cancer and Blood Services, Auckland City Hospital, Auckland, New Zealand; 3 School of Chemical Sciences, Faculty of Science, University of Auckland, Auckland, New Zealand

## Abstract

This study evaluated the impact of calcium and magnesium on the *in vitro* degradation and *in vivo* clearance of oxaliplatin. Intact oxaliplatin and Pt(DACH)Cl_2_ were measured in incubation solutions by HPLC-UV. A clinical study determined changes in plasma concentrations of calcium and magnesium in cancer patients and their impact on oxaliplatin clearance. Kinetic analyses modelled oxaliplatin degradation reactions *in vitro* and contributions to oxaliplatin clearance *in vivo*. Calcium and magnesium accelerated oxaliplatin degradation to Pt(DACH)Cl_2_ in chloride-containing solutions *in vitro*. Kinetic models based on calcium and magnesium binding to a monochloro-monooxalato ring-opened anionic oxaliplatin intermediate fitted the *in vitro* degradation time-course data. In cancer patients, calcium and magnesium plasma concentrations varied and were increased by giving calcium gluconate and magnesium sulfate infusions, but did not alter or correlate with oxaliplatin clearance. The intrinsic *in vitro* clearance of oxaliplatin attributed to chloride-, calcium- and magnesium-mediated degradation predicted contributions of <2.5% to the total *in vivo* clearance of oxaliplatin. In conclusion, calcium and magnesium accelerate the *in vitro* degradation of oxaliplatin by binding to a monochloro-monooxalato ring-opened anionic intermediate. Kinetic analysis of *in vitro* oxaliplatin stability data can be used for *in vitro* prediction of potential effects on oxaliplatin clearance *in vivo*.

## Introduction

Calcium and magnesium have been implicated in the mechanism and clinical prevention of oxaliplatin-induced neurotoxicity. Oxaliplatin (((1 R,2 R)-cyclohexane-1,2-diamine)(ethanedioato-O,O’)platinum(II)) is an important anticancer drug for the treatment of many cancers of the gastrointestinal tract^1–8^. It, in combination with a fluoropyrimidine, has become the standard chemotherapy for colorectal cancer in both adjuvant and palliative settings^1, 4–7^. However, its neurotoxicity is a major treatment-limiting adverse effect resulting in a dose reduction or early termination of the drug treatment and long term toxicity that may adversely affect patient’s quality of life for many months to years^9^. Clinically, most patients undergoing oxaliplatin therapy typically experience cold-induced peripheral paraesthesia, and some experience muscle cramps, throat or jaw tightness, and pharyngolaryngeal dysesthesia immediately after the oxaliplatin infusion^9^. Abnormal spontaneous high-frequency motor unit action potentials are detectable on electromyography, reflecting hyper-excitability of these peripheral nerves^10–12^. The mechanism of this acute neurotoxicity of oxaliplatin has been proposed to be related to the prolonged opening of calcium-dependent voltage-gated sodium channels resulting from chelation of calcium by oxalate^13–16^. The prolonged opening of these sodium channels may induce cellular stress, which in addition to the accumulation of platinum compounds in the dorsal root ganglial cells, may cause damage to cell body and axon^17–19^, and contribute to the development of chronic peripheral sensory neurotoxicity of oxaliplatin. It has also been suggested that oxaliplatin and/or its metabolites may increase calcium influx into the cytosol of peripheral neurons, which in turn may enhance the activation of sodium and potassium channels, calcium regulated transcription factors and intracellular signalling involving calcium-dependent protein kinases^20, 21^. On the basis of a retrospective clinical study suggesting neuroprotective effects of calcium gluconate and magnesium sulfate (CaGluc/MgSO_4_) infusions given concurrently with oxaliplatin therapy^22^ and supporting preclinical data^23^, these infusions became routinely used without further confirmatory prospective randomised trials or evaluation of any potential pharmacokinetic interactions with oxaliplatin. Since 2013, after two prospective randomised trials definitively showing a lack of neuroprotective effects of these infusions against both acute and chronic forms of oxaliplatin neurotoxicity^24, 25^, they are now commonly omitted from the routine clinical practice^26^.

**Figure 1:**
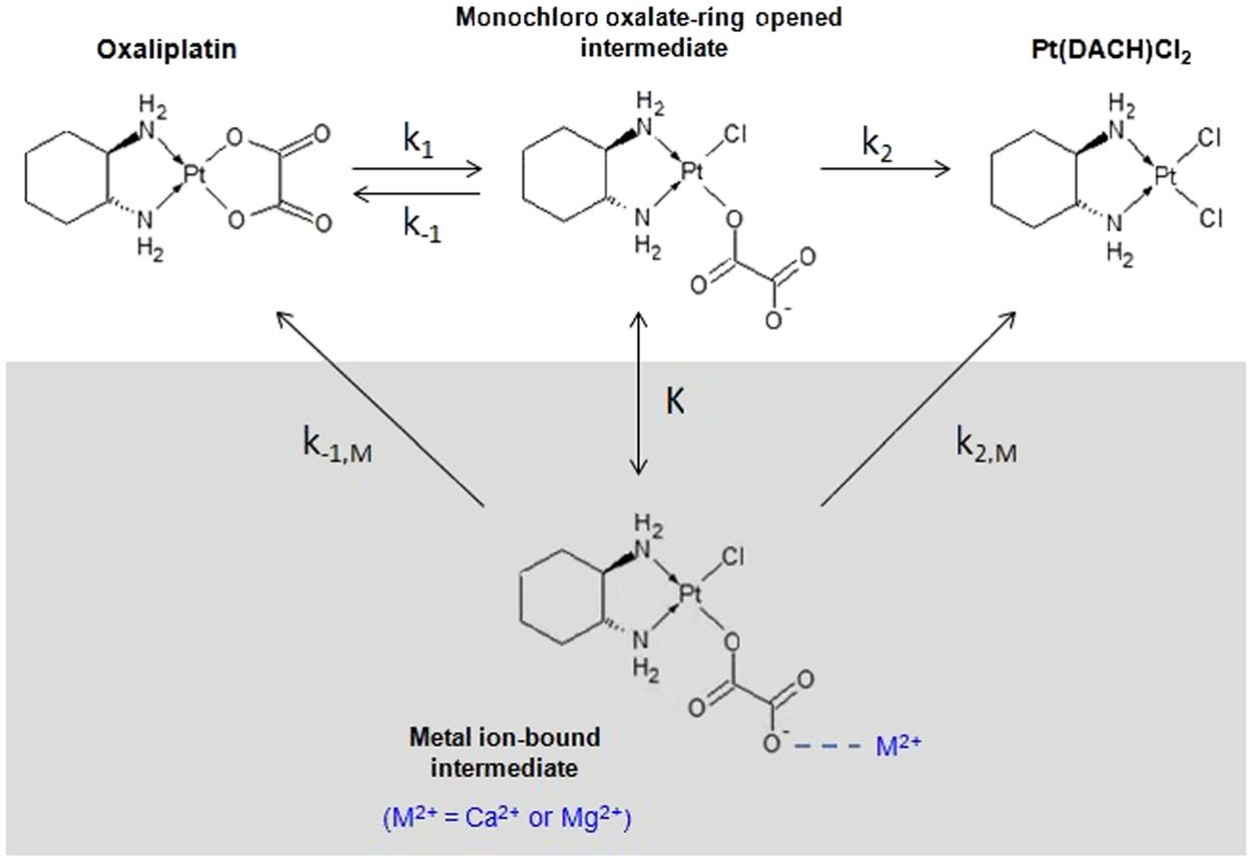
Reaction scheme of oxaliplatin degradation in chloride containing solution and chemical structures of intact oxaliplatin and its degradation products. A proposed additional reaction in CaCl_2_ and MgCl_2_ is shown in the shaded area. K, k: rate constants. M: metal.

The potential impact of calcium and magnesium on the *in vitro* degradation and *in vivo* clearance of oxaliplatin was previously unknown. Once administered oxaliplatin has been proposed to undergo rapid non-enzymatic biotransformation reactions with water and nucleophiles such as chloride, methionine and glutathione^27–29^. It is not known to be subject to CYP-P450 mediated metabolism. The initial reaction in oxaliplatin biotransformation can involve displacement of the oxalate group by water and chloride, resulting in formation of reactive species such as monochloro-, dichloro-, and diaquo-DACH platinum, where DACH is the common abbreviation for cyclohexane-1,2-diamine^29^. Initial degradation of oxaliplatin in the presence of chloride *in vitro* was previously suggested to lead to formation of an intermediate species, monochloro-monooxalato ring-opened complex, [Pt(DACH)oxCl]^−^^30^ (Fig. 1). This complex can convert back to oxaliplatin or transform into the final product, Pt(DACH)Cl_2_, resulting in overall slow degradation of oxaliplatin. The contributions made by these different ligand displacement reactions to the *in vivo* clearance of oxaliplatin are not well understood. To date, there have been few previous attempts to apply *in vitro – in vivo* extrapolation methods to predicting effects on the *in vivo* clearance of oxaliplatin. In drug discovery, techniques for predicting *in vivo* clearance of drugs using *in vitro* drug metabolism kinetic data has been used, particularly *in vitro* hepatocyte or microsomal metabolic stability kinetic data for predicting hepatic clearance of drugs *in vivo* ^31, 32^. For drugs, such as oxaliplatin, that are not metabolised by the liver, there is little literature available about the prediction of *in vivo* clearance using *in vitro* kinetic data.

With this background, we sought to evaluate the potential impact of calcium and magnesium on the *in vitro* degradation and the *in vivo* clearance of oxaliplatin. Drug stability studies and kinetic modelling were used to explore reactions of oxaliplatin and its degradation products with calcium and magnesium *in vitro*. A clinical study was undertaken to determine changes in plasma concentrations of calcium and magnesium in cancer patients given oxaliplatin with or without infusions of calcium gluconate and magnesium sulfate, and their impact on oxaliplatin clearance. These *in vitro* and clinical datasets provided an opportunity to develop and exemplify experimental approaches for prediction of effects on oxaliplatin clearance *in vivo* from oxaliplatin stability data generated *in vitro*.

## Results

### *In vitro* degradation of oxaliplatin

**Figure 2:**
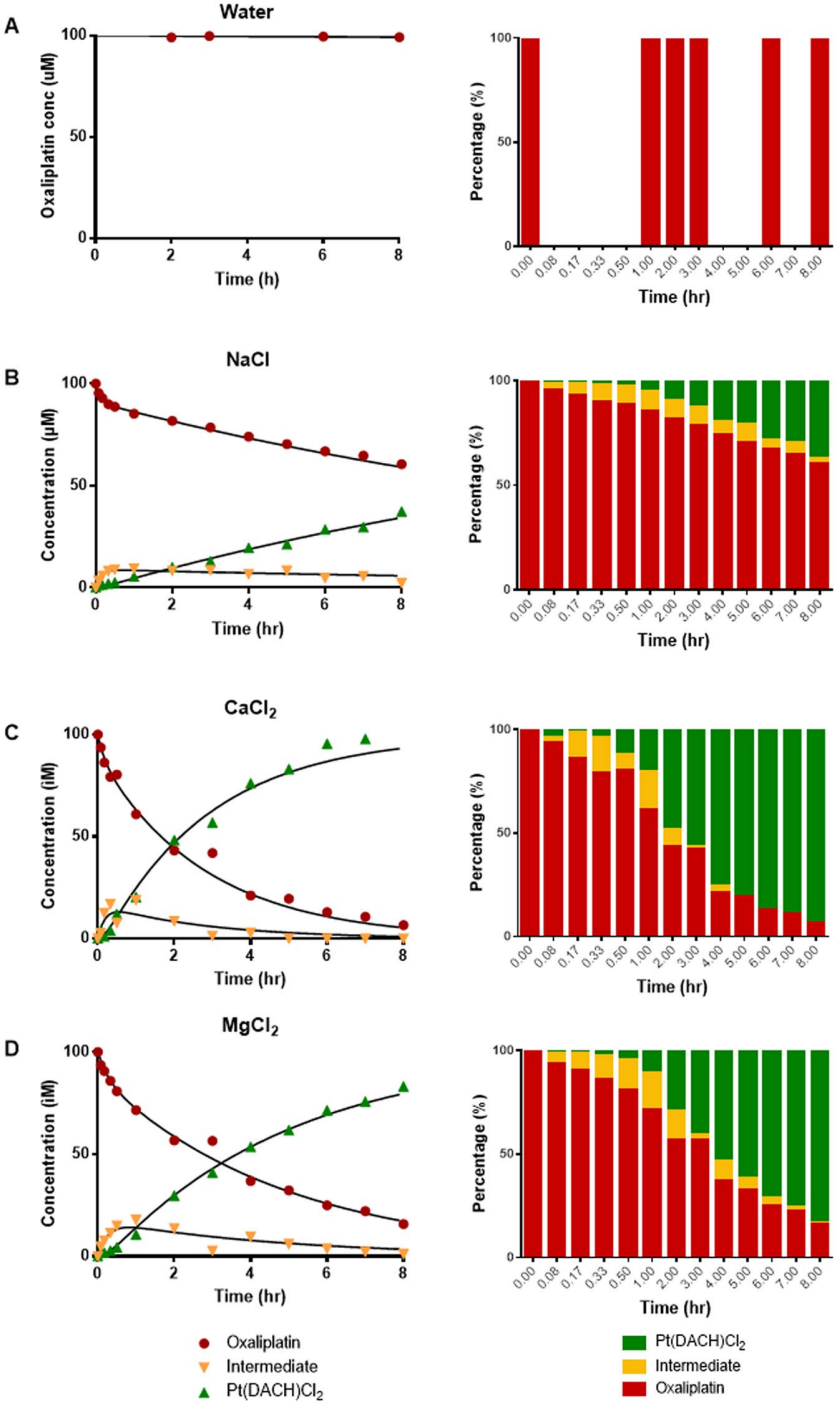
Time-course and mass balance analysis of oxaliplatin degradation in (**A**) water, (**B**) 150 mM NaCl, (**C**) 75 mM CaCl_2_, and (**D**) 75 mM MgCl_2_ solutions under physiological conditions (pH 7.3; 37 °C). Oxaliplatin was unstable in chloride containing solutions in contrast to its stability in water. Oxaliplatin degradation was accelerated in the presence of calcium and magnesium. Red: Intact oxaliplatin. Yellow: intermediate. Green: Pt(DACH)Cl_2_. Data points represent the mean of independent replicate measured values. The lines represent kinetic models shown in the text whose rate constants are shown in Table 1.

To evaluate the potential impact of calcium and magnesium on the *in vitro* degradation of oxaliplatin, oxaliplatin was incubated in water and solutions containing NaCl, CaCl_2_ and MgCl_2_ at 37 °C for 8 hours. Incubation samples were collected and analysed using a validated HPLC-UV method^33^ to determine oxaliplatin and Pt(DACH)Cl_2_ concentrations at pre-defined time points over the incubation period. Data were presented as plots of concentration versus time and the mass balance of the reaction (Fig. 2). Oxaliplatin was unstable in the presence of chloride and degraded to Pt(DACH)Cl_2_ via an intermediate species (Fig. 1), as previously shown by Jerremalm *et al*.^30^ who had identified the intermediate to be [Pt(DACH)oxCl]^−^. In contrast, oxaliplatin remained stable in water (Fig. 2). The rate of oxaliplatin degradation to Pt(DACH)Cl_2_ was accelerated in the presence of calcium and magnesium ions in association with higher transient formation of the intermediate species than was observed in NaCl.

### Kinetic analysis of *in vitro* degradation

The kinetic model proposed by Jerremalm *et al*.^29, 30^ for the stepwise reaction of oxaliplatin with chloride, comprising of a reversible first step producing [Pt(DACH)oxCl]^−^ followed by an irreversible second step producing Pt(DACH)Cl_2_ (Fig. 1), was fitted to the mean measured concentration values obtained at time points during the *in vitro* degradation studies. Rate constants (Table 1) were obtained by least-squares fitting to the analytical expressions shown below (Equations (1–5) for the reaction scheme in Fig. 1 to the three species simultaneously^34^. Standard errors for the rate constants were derived from a Jack-knife procedure^35^.

1 $${\lambda }_{1}=\frac{({k}_{1}+{k}_{-1}+{k}_{2})+\sqrt{{({k}_{1}+{k}_{-1}+{k}_{2})}^{2}-4{k}_{1}{k}_{2}}}{2}$$

2 $${\lambda }_{2}=\frac{({k}_{1}+{k}_{-1}+{k}_{2})-\sqrt{{({k}_{1}+{k}_{-1}+{k}_{2})}^{2}-4{k}_{1}{k}_{2}}}{2}$$

3 $$[Oxaliplatin]={[Oxaliplatin]}_{0}\{\frac{{k}_{1}({\lambda }_{1}-{k}_{2})}{{\lambda }_{1}({\lambda }_{1}-{\lambda }_{2})}{e}^{-{\lambda }_{1}t}+\frac{{k}_{1}({k}_{2}-{\lambda }_{2})}{{\lambda }_{2}({\lambda }_{1}-{\lambda }_{2})}{e}^{-{\lambda }_{2}t}\}$$

4 $$[Int]={[Oxaliplatin]}_{0}\{\frac{-{k}_{1}}{({\lambda }_{1}-{\lambda }_{2})}{e}^{-{\lambda }_{1}t}+\frac{{k}_{1}}{({\lambda }_{1}-{\lambda }_{2})}{e}^{-{\lambda }_{2}t}\}$$

5 $$[Pt(DACH)C{l}_{2}]={[Oxaliplatin]}_{0}\{\frac{{k}_{1}{k}_{2}}{{\lambda }_{1}{\lambda }_{2}}+\frac{{k}_{1}{k}_{2}}{{\lambda }_{1}({\lambda }_{1}-{\lambda }_{2})}{e}^{-{\lambda }_{1}t}-\frac{{k}_{1}{k}_{2}}{{\lambda }_{2}({\lambda }_{1}-{\lambda }_{2})}{e}^{-{\lambda }_{2}t}\}$$

**Table 1:** Rate constants (±standard error) for oxaliplatin degradation reactions in NaCl (150 mM), CaCl_2_ (75 mM) and MgCl_2_ (75 mM) at 37 °C.

(hr^−1^)	k_1_	k−_1_	k_2_	λ_1_	λ_2_
NaCl	0.726 ± 0.082	6.9 ± 1.1	0. 605 ± 0.052	8.2 ± 1.2	0.054 ± 0.002
CaCl_2_	0.84 ± 0.27	2.6 ± 2.3	2.22 ± 0.63	5.3 ± 3.0	0.353 ± 0.028
MgCl_2_	0.625 ± 0.071	2.10 ± 0.75	1.23 ± 0.18	3.74 ± 0.98	0.205 ± 0.007

The parameters λ_1_ and λ_2_ are the observed biexponential decay constants, and are combinations of the rate constant for oxalate ring opening k_1_, the rate constant for ring closing k_−1_, and the rate constant for oxalate loss k_2_. The kinetic models fitted the experimental data for all conditions (Fig. 2). Addition of CaCl_2_ or MgCl_2_ increased the observed rate of degradation of oxaliplatin and caused increased amounts of the intermediate species to form. However, analysis of the data showed that the k_1_ rate constants for the oxalate ring opening step in the oxaliplatin degradation reaction were similar in 150 mM NaCl, 75 mM CaCl_2_ and 75 mM MgCl_2_, while the k_−1_ rate constants for the *back* reaction corresponding to oxalate chelate ring closing and reformation of oxaliplatin from the [Pt(DACH)oxCl]^−^ intermediate were decreased in 75 mM CaCl_2_ and 75 mM MgCl_2_ as compared to 150 mM NaCl. Finally, the values for the k_2_ rate constant for the loss of the monodentate oxalate ligand from [Pt(DACH)oxCl]^−^ leading to formation of Pt(DACH)Cl_2_ were increased in 75 mM CaCl_2_ and 75 mM MgCl_2_ as compared to 150 mM NaCl. Thus, the observed increase in degradation is not due to an increased rate of the initial oxalate ring opening, but rather is due to a combination of a decrease in the reverse rate of oxalate ring closure combined with an increase in the rate of loss of oxalate.

These findings suggested that calcium and magnesium did not interact significantly with oxaliplatin at the concentrations studied, but bound to the monodentate oxalate-containing complex [Pt(DACH)oxCl]^−^, and in doing so, decreased the rate of oxalate ring closure and increased the rate of loss of oxalate from the ring-opened intermediate. The kinetics of the reaction mechanism were therefore modelled further by including an additional metal bonded intermediate species as shown in the shaded area of Fig. 1. With the assumption that the rate constant for the reformation of oxaliplatin from the metal bonded intermediate (k_−1,M_) was zero, then the calculated values of the equilibrium constant (K) for the reversible conversion of [Pt(DACH)oxCl]^−^ to the metal bonded intermediate were 20 M^−1^ for Ca^2+^ and 30 M^−1^ for Mg^2+^. If k_−1,M_ was not zero then the equilibrium constants would be higher. These equilibrium constant values indicate that in 75 mM CaCl_2_ or MgCl_2_ 60% or 70% respectively of the [Pt(DACH)oxCl]^−^ is ion-paired with the metal. Using these equilibrium constant values, the values of the rate constant for the conversion of the metal bonded intermediate to Pt(DACH)Cl_2_ (k_2,M_) were calculated to be 3.3 hr^−1^ and 1.5 hr^−1^ for calcium and magnesium, respectively.

### Plasma calcium and magnesium concentrations after CaGluc/MgSO_4_ infusions in patients

**Figure 3:**
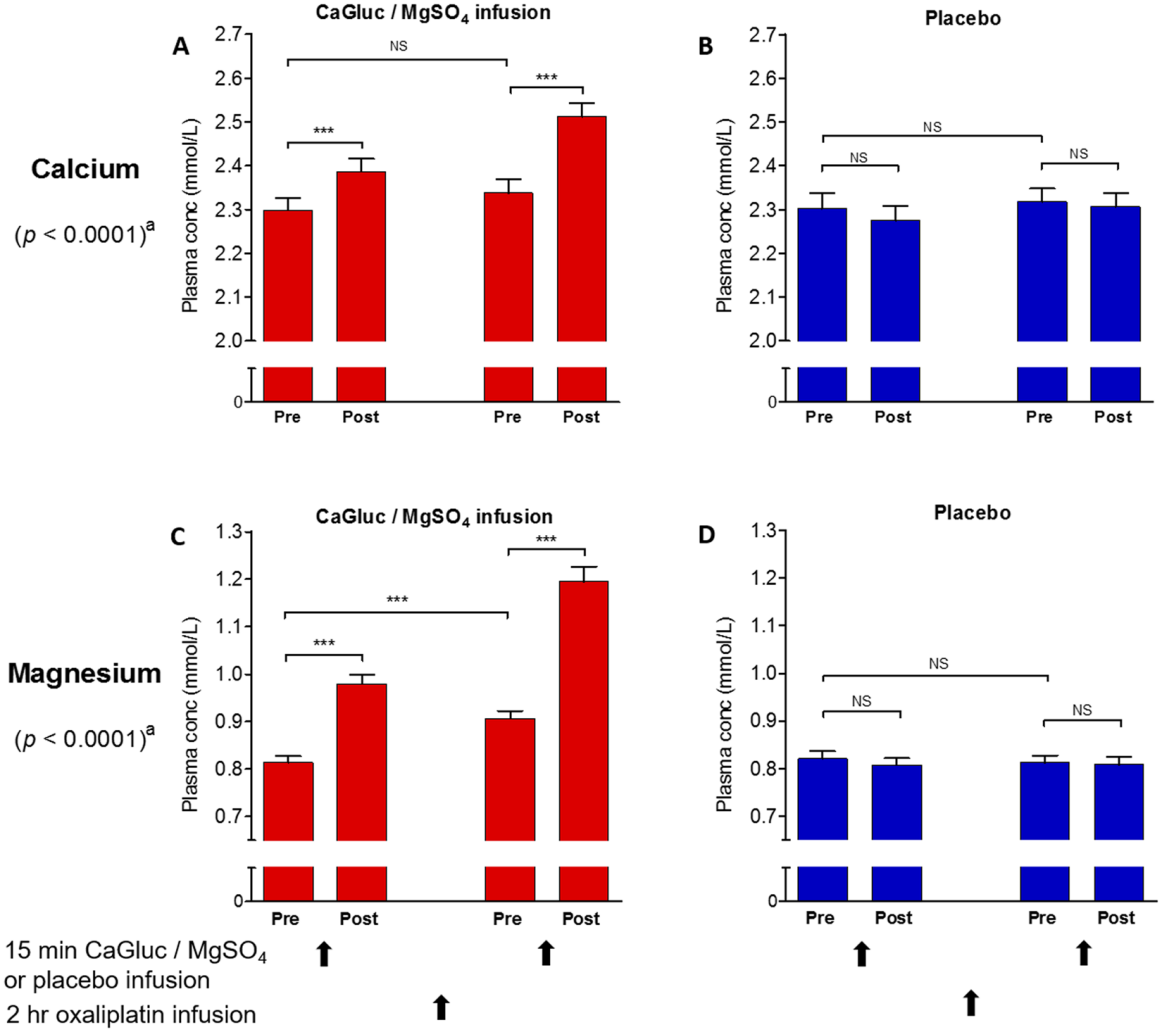
Plasma calcium (**A**,**B**) and magnesium (**C**,**D**) concentrations in colorectal cancer patients measured before and after CaGluc/MgSO_4_ (red) or placebo (blue) infusions given with oxaliplatin chemotherapy. “Pre” and “post”refer to the timing of the plasma calcium and magnesium measurements before or after the CaGluc/MgSO_4_ infusions. The p values shown as *p* < 0.0001 are for a comparison of measurements determined with CaGluc/MgSO_4_ versus placebo infusions using repeated measures one way ANOVA, and those shown as *** (*p* < 0.001) or NS (not significant) are from Tukey’s multiple comparison test. Plasma calcium and magnesium concentrations were increased after CaGluc/MgSO_4_ infusions but not after placebo infusions. CaGluc/MgSO_4_: calcium gluconate/magnesium sulfate infusions.

The *in vitro* findings described above suggested that calcium and magnesium may alter the *in vivo* clearance of oxaliplatin by accelerating its degradation via binding to [Pt(DACH)oxCl]^−^. Plasma calcium and magnesium concentrations vary between patients, on different occasions, and are altered in the presence of cancer, associated disease processes and treatments^36, 37^. Until recently, CaGluc/MgSO_4_ infusions had been routinely given concurrently with oxaliplatin for the purpose of limiting neurotoxicity^26^ but their effects on oxaliplatin clearance or plasma calcium and magnesium levels had not been well studied. In a randomised placebo-controlled crossover clinical study, we sought to understand how variation in plasma calcium and magnesium concentrations, and the administration of CaGluc/MgSO_4_ infusions, influenced oxaliplatin clearance in cancer patients receiving chemotherapy, by measuring plasma concentrations of calcium and magnesium before and after CaGluc/MgSO_4_ and placebo infusions given with oxaliplatin. We previously reported that oxaliplatin clearance was not altered by giving CaGluc/MgSO_4_ infusions^24^. The mean oxaliplatin clearance was 35.3 L/hr (SD 9.8) with CaGluc/MgSO_4_ infusions and 33.6 L/hr (7.7) with placebo infusions (*p* = 0.17). Here we report that plasma concentrations of calcium and magnesium were significantly higher after the first CaGluc/MgSO_4_ infusions than at baseline (calcium 1.04-fold increase, *p* < 0.001; magnesium 1.2-fold increase, *p* < 0.001) (Fig. 3). Plasma calcium concentration had returned to near baseline levels immediately prior to the second CaGluc/MgSO_4_ infusion two hours later but increased again after the second infusion (1.09-fold increase, *p* < 0.001). Plasma magnesium concentration, however, remained significantly higher (1.13-fold increase, *p* < 0.001) than baseline when the second infusion was due and were increased further following the second infusion (1.5-fold increase, *p* < 0.001). There were no significant changes in plasma calcium and magnesium levels after the placebo infusions.

**Figure 4:**
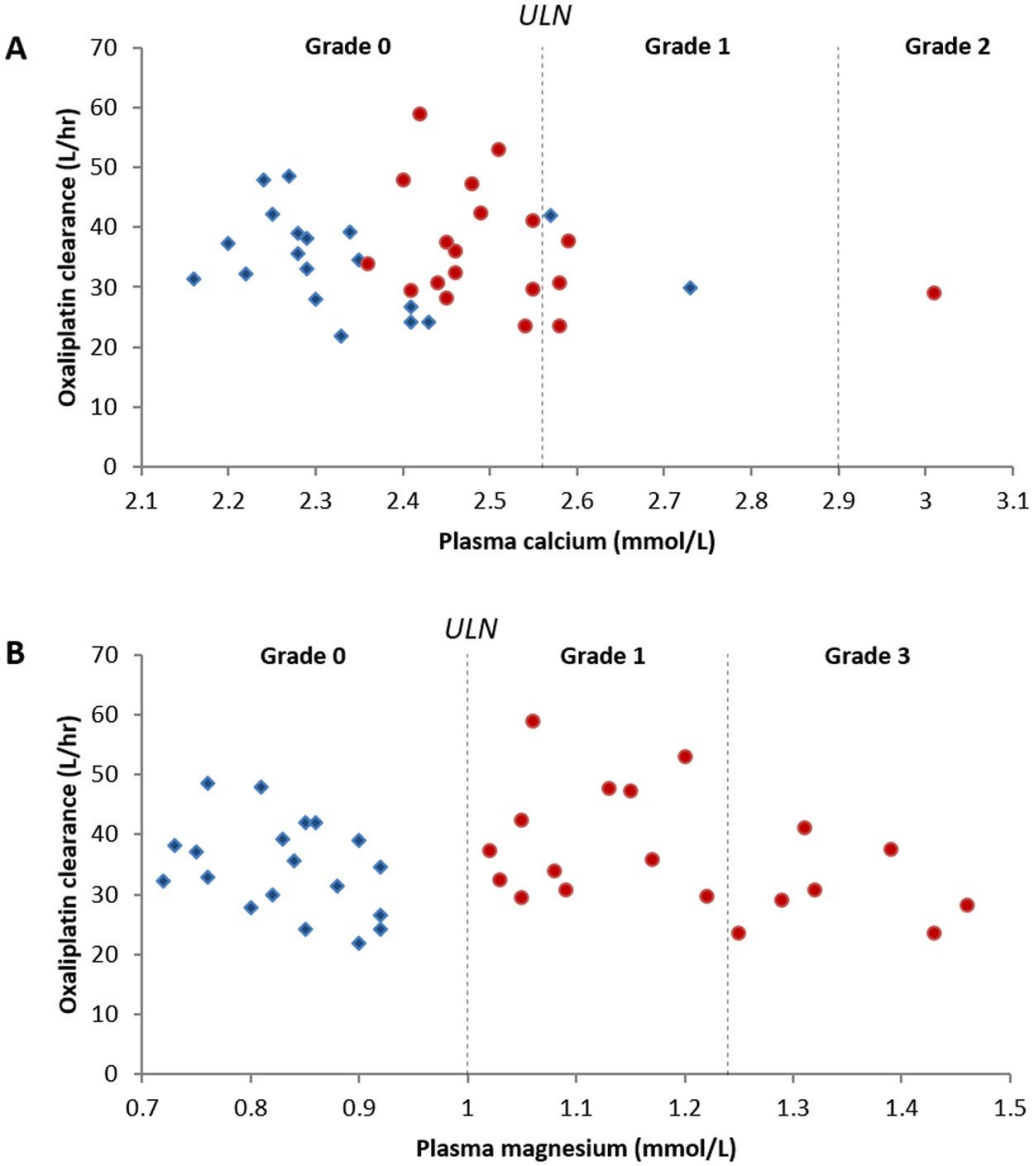
Correlation plots of oxaliplatin clearance versus plasma calcium (**A**) and magnesium (**B**) concentrations in colorectal cancer patients. Patients (n = 19) received CaGluc/MgSO_4_ and placebo infusions on alternate cycles of oxaliplatin treatment in random order, during which oxaliplatin clearance and plasma calcium and magnesium levels were measured. Data points represent oxaliplatin clearance values and the maximal plasma calcium and magnesium concentrations achieved during the placebo (blue) and CaGluc/MgSO_4_ infusion cycle (red) for each patient. Oxaliplatin clearance did not correlate with plasma calcium or magnesium concentrations (correlation coefficient = −0.38; r^2^ = 0.14; and *p* = 0.11). Vertical lines show Common Toxicity Criteria Adverse Event (CTCAE) severity gradings for hypercalcaemia and hypermagnesaemia. ULN: upper limit of normal.

Oxaliplatin clearance values measured on treatment cycles given with CaGluc/MgSO_4_ or placebo infusions were plotted against their corresponding maximal plasma calcium and magnesium levels for each study patient (Fig. 4). Maximum plasma concentrations of calcium and magnesium were within normal limits after placebo infusions. After CaGluc/MgSO_4_ infusions, however, maximum plasma levels exceeded the upper limit of normal in 4 (21%) and 19 (100%) patients for calcium and magnesium, respectively. According to Common Toxicity Criteria Adverse Effect version 4.0 grading, elevated plasma calcium were severity grade 1 in 3 patients (16%) and grade 2 in 1 patient (5%), while elevated plasma magnesium was severity grade 1 in 12 patients (63%) and grade 3 in 7 patients (37%). Pearson co-efficient correlation analysis showed that oxaliplatin clearance did not correlate with plasma calcium (*p* = 0.33) or magnesium (*p* = 0.60) concentrations.

### *In vitro* Prediction of calcium-, magnesium- and chloride- mediated clearance of oxaliplatin

To estimate potential contributions of calcium-, magnesium- and chloride-mediated degradation to the *in vivo* clearance of oxaliplatin in patients, an *in vitro – in vivo* extrapolation method was developed. First, the *in vitro* intrinsic clearances of oxaliplatin attributable to calcium-, magnesium- and chloride-mediated degradation were calculated. Non-compartmental analysis was used to calculate the AUC_0−infinity_ for oxaliplatin concentration versus time for each experimental condition. Then the total *in vitro* clearance of oxaliplatin for each condition was obtained by dividing the amount of oxaliplatin added to the solution per unit volume by the AUC_0−infinity_. Oxaliplatin clearance attributable to calcium- or magnesium- mediated degradation was then calculated by subtracting the clearance attributable to chloride from the total *in vitro* clearance value for the relevant experimental condition. Calculated values for oxaliplatin clearance attributable to chloride, calcium and magnesium were then plotted against the concentrations of the relevant ion in the incubation solution and analysed by linear regression. The *in vitro* intrinsic clearance attributable to chloride, calcium and magnesium was taken from the slope of the linear regression fit to its data as shown in Table 2 and Supplementary Figure. These data were then scaled to the *in vivo* setting by calculating the total extracellular fluid (ECF) content of calcium, magnesium and chloride from estimates of ECF volume and measured plasma concentrations of these ions in patients from our clinical study. *In vivo* clearance predictions were then made by multiplying the *in vitro* intrinsic clearance value by the calculated ECF content value of each ion.

**Table 2:** Calculation of the *in vitro* intrinsic clearance of oxaliplatin attributable to chloride-, calcium-, and magnesium-mediated oxaliplatin degradation.

Incubation solution	Conc. of solution (mM)	Amount of reactant^a^ (mmol)	Amount of chloride (mmol)	Oxaliplatin AUC_0−infinity_ (μmol/L*h)	Total *in vitro* oxaliplatin clearance^b^ (L/h)	Oxaliplatin clearance attributable to calcium and magnesium^c^ (L/h)
NaCl	15		15	17243.5	0.0058	
	50		50	5769.7	0.0173	
	150		150	1809.1	0.0553	
CaCl_2_	1.8	1.8	150	1201.2	0.0832	0.0280
	37.5	37.5	150	563.3	0.1775	0.1222
	75	75	150	281.9	0.3547	0.2994
MgCl_2_	1.8	1.8	150	1333.0	0.0750	0.0197
	37.5	37.5	150	756.5	0.1322	0.0769
	75	75	150	428.8	0.2332	0.1780

The *in vitro* learance of oxaliplatin was calculated by non-compartmental analysis of oxaliplatin concentration versus time data from *in vitro* incubations with different concentrations of each ion solution. The amount of oxaliplatin added to the incubation solutions was 100 µmol.

^a^Reactant: calcium ions in CaCl_2_, magnesium ions in MgCl_2_. ^b^Total oxaliplatin clearance as calculated by non-compartmental pharmacokinetic analysis: clearance = amount of oxaliplatin per unit volume/AUC_0-infinity_. ^c^Oxaliplatin clearance attributable to chloride, calcium and magnesium: Total *in vitro* oxaliplatin clearance minus the clearance attributable to 150 mmol of chloride (0.0553).

The *in vitro* intrinsic clearance of oxaliplatin attributable to calcium-, magnesium- and chloride-mediated degradation was calculated to be 0.0039, 0.0023 and 0.00037 L/hr/mmol, respectively (Table 3). The predicted *in vivo* oxaliplatin clearances attributable to calcium, magnesium and chloride were 0.15 L/hr, 0.03 L/hr and 0.68 L/hr, respectively, before CaGluc/MgSO_4_ infusions. After the CaGluc/MgSO_4_ infusion, these predicted *in vivo* oxaliplatin clearance values increased slightly to 0.16 L/hr for calcium and 0.05 L/hr for magnesium but remained unchanged for chloride. The combined oxaliplatin clearance attributable to the sum of calcium-, magnesium- and chloride-mediated degradation was predicted to be 0.86 and 0.89 L/hr before and after CaGluc/MgSO_4_ infusions, respectively. Considering the total *in vivo* clearance of oxaliplatin measured in our clinical study (35.3 L/hr), calcium-, magnesium- and chloride-mediated degradation was predicted to account for less than 2.5% of the total clearance of oxaliplatin *in vivo*.

**Table 3:** *In vitro* prediction of effects of calcium-, magnesium- and chloride-mediated oxaliplatin degradation on the *in vivo* clearance of oxaliplatin.

	*In vitro* intrinsic clearance	Scaling factors		*In vivo* clearance prediction	
	(L/hr/mmol)	Plasma concentration (mmol/L)	Extracellular Fluid content (mmol)	(L/hr)	(%)^a^
Calcium	0.0039	2.30/2.51^b^	38.1/41.6^b^	0.15/0.16^b^	0.4/0.5^b^
Magnesium	0.0023	0.81/1.19^b^	13.5/19.7^b^	0.03/0.05^b^	0.1/0.1^b^
Chloride	0.00037	104.4	1840	0.68	1.9
Combined	0.00657	—	—	0.86/0.89^b^	2.4/2.5^b^

^a^Percentage of measured *in vivo* oxaliplatin clearance (35.3 L/hr); ^b^Before/after CaGluc/MgSO_4_ infusions.

## Discussion

This study showed that calcium and magnesium accelerate oxaliplatin degradation by binding to the monochloro-monooxalato ring-opened anionic oxaliplatin intermediate, [Pt(DACH)oxCl]^−^, that forms in chloride-containing physiological solutions *in vitro*. To our knowledge, calcium and magnesium have not been previously reported to alter oxaliplatin degradation or bind to its degradation intermediates. We showed that the *in vitro* degradation of oxaliplatin to Pt(DACH)Cl_2_ was faster in the presence of calcium and magnesium by directly quantifying both intact oxaliplatin and Pt(DACH)Cl_2_ using HPLC-UV. Mass balance analysis revealed a deficit after accounting for these two compounds consistent with the transient formation of an intermediate species. Further evidence came from kinetic analyses and rate constants calculated for the reaction scheme in Fig. 1 suggesting that calcium and magnesium interacted with the oxalate ring-opened anionic oxaliplatin intermediate, [Pt(DACH)oxCl]^−^, resulting in a decreased rate of oxalate ring closure, and an increased loss of monodentate oxalate leading to formation of Pt(DACH)Cl_2_. Oxaliplatin was unstable in the presence of chloride as previous reported by Jerremalm *et al*.^30^ but our findings also provide a mechanism of how calcium and magnesium influence this process of oxaliplatin degradation. This new information may have important implications for understanding how oxaliplatin behaves under *in vivo* conditions when other reactants or cations are present.

This current study provides a new experimental approach for predicting effects on the *in vivo* clearance of oxaliplatin from *in vitro* studies of oxaliplatin degradation. It is, to our best knowledge, the first time that *in vitro – in vivo* extrapolation methods have been applied to predicting effects on oxaliplatin clearance. The concept was based on methods which are already established for *in vitro - in vivo* prediction of drug clearance mediated by hepatic microsomal metabolism by CYP-P450 and other enzymes, and which are commonly used in pre-clinical studies of the metabolic stability of new chemical entities^32^. However, oxaliplatin is not subject to hepatic metabolism, but degrades via leaving group displacement reactions, although little is known about their contributions to the *in vivo* clearance of oxaliplatin. To estimate effects on the *in vivo* clearance of oxaliplatin, we first obtained experimentally determined values for the *in vitro* intrinsic clearance of oxaliplatin attributable to calcium-, magnesium- and chloride-mediated oxaliplatin degradation, calculated from kinetic analyses of *in vitro* oxaliplatin stability data. Then, scaling factors were used to estimate the contributions of these processes to the *in vivo* clearance of oxaliplatin. This experimental approach has potential for providing new insights into mechanisms of oxaliplatin clearance. Previously, for example, it was suggested that oxaliplatin may initially react with water or chloride during its *in vivo* biotransformation^28, 29^. However, we found oxaliplatin to be stable in water *in vitro*, and that chloride-mediated oxaliplatin degradation was predicted to contribute only 1.9% to the total *in vivo* clearance of oxaliplatin. This stability of oxaliplatin in pure water or in chloride may be due to slow oxalate ring opening and/or fast oxalate ring closing. These findings suggest that oxaliplatin degradation mediated only by water or chloride may contribute less than previously thought to the total clearance of oxaliplatin *in vivo*, as suggested by a low formation of Pt(DACH)Cl_2_ from oxaliplatin under *in vivo* conditions^24, 38^.

In cancer patients receiving oxaliplatin chemotherapy, we found that plasma calcium and magnesium concentrations were increased significantly after the infusions of calcium gluconate and magnesium sulfate given immediately before and after oxaliplatin chemotherapy. These infusions were, until recently, routinely used in the clinic for the purpose of reducing neurotoxicity. Statistically significant increases in plasma calcium and magnesium occurred following each of the two CaGluc/MgSO_4_ infusions. Post-infusion elevations in plasma calcium were CTCAE severity grade 1 in three patients and grade 2 in one patient, while elevations in plasma magnesium were CTCAE grade 1 in twelve patients and grade 3 in seven patients. There has not been a previous study, which we are aware of, showing these effects on plasma calcium and magnesium levels in patients immediately or soon after these infusions prior to this current study. In our study, plasma calcium and magnesium levels were measured only at baseline, 20 minutes after the first CaGluc/MgSO_4_ infusions and immediately after the second CaGluc/MgSO_4_ infusion. We did not fully evaluate the time-course, duration, extent or clinical safety concerns related to these treatment-associated elevations in plasma calcium and magnesium levels. Previously, Gamelin *et al*.^39^ reported no difference in plasma calcium and magnesium levels after the end of oxaliplatin infusion given without CaGluc/MgSO_4_ infusions. Their reported values were very similar to those found in the current study at baseline or after placebo infusions. This clinical study also provided an opportunity to explore potential relationships between plasma calcium and magnesium levels and the clearance of oxaliplatin. Plasma calcium and magnesium varied widely between different patients and treatment cycles but did not correlate with oxaliplatin clearance. This finding was in keeping with the *in vitro* prediction that calcium- and magnesium-mediated degradation contributed a relative small amount (<2.5%) to the total *in vivo* clearance of oxaliplatin.

The method we describe for the *in vitro* prediction of effects on the *in vivo* clearance of oxaliplatin could assist with translating new treatments to clinical evaluation for preventing oxaliplatin neurotoxicity. Many new candidate treatments for preventing neurotoxicity of oxaliplatin are being identified by preclinical studies, for example, L-type calcium channel blockers^20^ and calcium/calmodulin-dependent kinase inhibitors^21^. Our method could identify the potential for deleterious pharmacokinetic interactions with oxaliplatin prior to clinical studies. In this way, this *in vitro* assessment will complement trial design and endpoints we recently proposed for the early clinical evaluation of investigational treatments for preventing oxaliplatin neurotoxicity^40^.

In conclusion, calcium and magnesium accelerate the *in vitro* degradation of oxaliplatin by binding to a monochloro-monooxalato ring-opened anionic intermediate. Kinetic analyses of *in vitro* oxaliplatin stability data predicted the contributions of calcium-, magnesium- and chloride-mediated degradation to the total *in vivo* clearance of oxaliplatin in patients. *In vitro-in vivo* extrapolation methods can be used in future studies for the *in vitro* prediction of potential effects on oxaliplatin clearance *in vivo*.

## Materials and Methods

### *In vitro* incubation studies

Oxaliplatin and Pt(DACH)Cl_2_ were obtained from Sigma-Aldrich (St Louis, MO, USA). Hydroxyethylpiperazine-N’-2-ethane sulfonic acid (‘HEPES’) was obtained from Gibco-BRL Life Technologies (Grand Island, NY, USA). Powder sodium chloride (NaCl) and magnesium chloride (MgCl_2_) anhydrous were obtained from Sigma Aldrich (St Louis, MO, USA). Calcium chloride (CaCl_2_) was obtained from Riedel-de Haen AG (Germany). Methanol of chromatographic grade, triflic acid (98% reagent grade) was obtained from Sigma Aldrich (St Louis, MO, USA). All solutions and mobile phase were prepared using Milli-Q grade water (Millipore, Bedford, USA).

To study the stability of oxaliplatin in the presence of chloride, calcium and magnesium under physiological conditions, oxaliplatin (100 μM prepared in Milli-Q water) was incubated in water alone, NaCl (15, 50, and 150 mM), CaCl_2_ (1.8, 37.5 and 75 mM), and MgCl_2_ (1.8, 37.5 and 75 mM) in HEPES buffer at pH 7.3 and 37 °C. A temperature regulated water-bath (Julabo TW12, John Morris Scientific Ltd., Auckland) was used to maintain temperature during incubation. Samples for analysis were taken at 0, 5, 10, 20, 30, and 60 minutes then hourly thereafter until 8 hours. To detect oxaliplatin and its degradation product, Pt(DACH)Cl_2_, these incubation samples were analysed using a Hewlett Packard HP1200 HPLC online system. This included a binary pump, a degasser and an autosampler (Wilmington, DE, USA), a Waters µBondapak C_18_ 3.9 × 300 mm column (Waters, Massachusetts, USA) with a guard column (Phenomenex, Torrance, LA, USA). The UV detector used was Millipore Waters Lambda-max model 480 LC Spectrophometer (Millipore, Land Cove, Australia). The HPLC separations were performed at room temperature using a mobile phase containing 3% methanol in Milli-Q water (adjusted to pH 2.5 with triflic acid). The flow rate was 0.5 mL/min. The UV wavelength monitored was 210 nm with a reference of 550 nm. The injection volume of all samples was 50 µL. Data acquisition and processing were performed using HP4500 ChemStation and HP1200 Agilent ChemStation offline software B.04.01 (Agilent Technologies, Avodale, USA). The samples were analysed immediately after they were taken from the incubation solutions whenever possible, otherwise they were snap frozen using liquid nitrogen and thawed within a minute before being injected onto the HPLC for analysis to avoid continuation of degradation of oxaliplatin at higher temperatures. All samples from each incubation study were analysed on the same day using the same mobile phase. Concentrations of oxaliplatin and Pt(DACH)Cl_2_ were determined by using their respective calibration curves to convert the areas under the chromatographic peaks to concentration values. The concentrations of the intermediate species were estimated from a mass balance after accounting for oxaliplatin and Pt(DACH)Cl_2_. The kinetics of degradation reactions were modelled as described below.

### Clinical study

In our previously reported clinical study^24^, a randomised double-blind placebo-controlled design was used to evaluate the effects of calcium gluconate (1 g) and magnesium sulfate (1 g) (CaGluc/MgSO_4_) infusions on the pharmacokinetics and acute neurotoxicity of oxaliplatin. Each patient undergoing oxaliplatin chemotherapy was given either CaGluc/MgSO_4_ or placebo infusions immediately before and after oxaliplatin infusion on cycle 1 then the opposite study infusion on cycle 2. This study was approved by the Northern Y Regional Ethics Committee (approval number NTY/11/01/005) and was conducted in accordance with its guidelines and regulations. Informed consent was obtained from all study participants. The plasma pharmacokinetic samples were collected at 13 predefined times including at baseline, during and 3 hours post oxaliplatin infusion. The samples used for this current study were taken at baseline, 20 minutes post first CaGluc/MgSO_4_ or placebo infusion, immediately prior and post second CaGluc/MgSO_4_ or placebo infusions. The plasma was immediately prepared by centrifugation at 4 °C and 5000 G for 5 minutes, then snap-frozen using liquid nitrogen. The samples were stored at −80 °C until analysis. Plasma calcium (albumin adjusted) and magnesium concentrations were measured using Cobas 8000 modular analyser (Roche Diagnostics Ltd., Switzerland) at LabPlus, Auckland City Hospital (Auckland, New Zealand). Data were presented as measured values and the levels of calcium and magnesium were graded using Common Toxicity Criteria Adverse Effect (CTCAE) version 4. Plasma calcium and magnesium concentrations were compared between baseline and post CaGluc/MgSO_4_ or placebo infusions by repeated measures one-way ANOVA analysis. Correlation between calcium and magnesium concentrations and observed oxaliplatin clearance for each patient obtained from our previous study^24^ was assessed by Pearson correlation analysis. All statistical analyses were performed using PRISM 6, GraphPad, San Diego, USA.

### Kinetic modelling

Kinetic analysis of oxaliplatin reactivity in the presence of calcium and magnesium was done using the mean concentration values for oxaliplatin and Pt(DACH)Cl_2_ at each sampling time. Rate constants were obtained by least-square fitting the analytical expressions for the reaction scheme to the three species simultaneously (Fig. 1 – unshaded area), using the Solver function in Microsoft Excel with the three rate constants k_1_, k_−1_, and k_2_ as adjustable parameters. Estimates of the standard errors for these parameters were then obtained using a Jack-knife procedure.

To determine *in vitro* intrinsic clearance of oxaliplatin attributable to chloride-, calcium- and magnesium-mediated degradation, the following steps were undertaken: (1) AUC_0−infinity_ for oxaliplatin concentration versus time for each *in vitro* incubation experimental condition was calculated using non-compartmental analysis; (2) total *in vitro* clearance of oxaliplatin for each condition was calculated by dividing the amount of oxaliplatin in the incubation solution per unit volume by the corresponding AUC_0−infinity_; (3) oxaliplatin clearance attributable to calcium- and magnesium-mediated degradation was calculated by subtracting the clearance attributable to chloride from the total *in vitro* clearance value for each condition; (4) calculated values for oxaliplatin clearance attributable to these ions were then plotted against the concentrations of the corresponding ions in the incubation solution and analysed by linear regression; and (5) finally, the *in vitro* intrinsic clearance attributable to chloride, calcium and magnesium was taken from the slope of the linear regression fit to its data (Table 2 and Supplementary Figure).

To extrapolate *in vitro* kinetic data from the incubation studies to *in vivo* oxaliplatin clearance attributable to plasma chloride, calcium and magnesium, a scaling factor was applied to the calculated intrinsic clearance for each of chloride, calcium and magnesium ions. The approach to scaling used in our study was a modified version of those used in the microsomal *in vitro-in vivo* extrapolation methods^32^. We used the estimated extracellular fluid (ECF) contents of chloride, calcium and magnesium in each patient. First, total body water was estimated as 60% of body weight in men and 50% in women, and the ECF volume was estimated as 40% of total body water. The ECF content of chloride, calcium and magnesium was then calculated by multiplying the measured plasma concentrations of these ions by the estimated ECF volume in each patient. Finally, *in vivo* clearance of oxaliplatin mediated by each of chloride, calcium and magnesium was calculated using the following formula: *in vivo* CL = *in vitro* CL_int_ x ECF content, where units for CL = L/h; CL_int_ = L/h/mmol; and ECF content = mmol).

## Electronic supplementary material

Supplementary Figure
