# Supplementary material for: Predicting effects on oxaliplatin clearance: in vitro, kinetic and clinical studies of calcium- and magnesium-mediated oxaliplatin degradation
Source: Sci Rep. 2017 Jun 22;7:4073. doi: 10.1038/s41598-017-04383-4 (PMC5481441; doi:10.1038/s41598-017-04383-4)

Predicting effects on oxaliplatin clearance: *in vitro,* kinetic and clinical studies of calcium- and magnesium-mediated oxaliplatin degradation

AUTHORS

Catherine H. Han[1, 2], Prashannata Khwaounjoo[1], Andrew G. Hill[1, 2], Gordon M. Miskelly[3], Mark J. McKeage[1, 2*]

1. Department of Pharmacology and Clinical Pharmacology and Auckland Cancer Society Research Centre, School of Medical Sciences, Faculty of Medical and Health Sciences, University of Auckland, Auckland, New Zealand.
2. Regional Cancer and Blood Services, Auckland City Hospital, Auckland, New Zealand.
3. School of Chemical Sciences, Faculty of Science, University of Auckland, Auckland, New Zealand

* Corresponding author

Supplementary Figure. Linear regression analysis of *in vitro* oxaliplatin clearance versus chloride, calcium and magnesium concentration.

Calculated values for *in vitro* oxaliplatin clearance attributable to chloride, calcium and magnesium were plotted against the concentrations of the relevant ion in the incubation solution and analysed by linear regression. The *in vitro* intrinsic clearance attributable to chloride, calcium and magnesium was taken from the slope of the linear regression fit to its data.

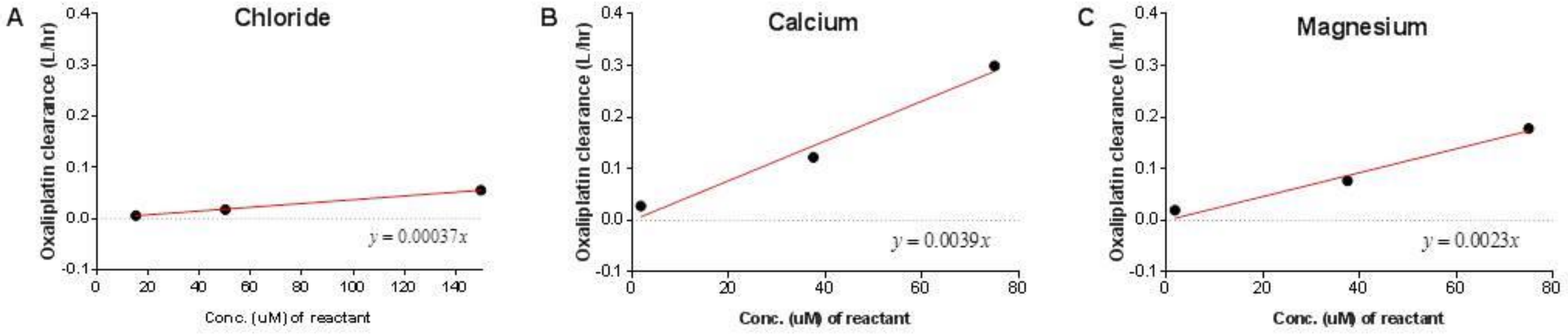

Supplement: Supplementary file 1 — Supplementary Figure [file 41598_2017_4383_MOESM1_ESM.pdf]
